# Exploring Anti-Breast Cancer Effects of Live *Pediococcus acidilactici* and Its Cell-Free Supernatant Isolated from Human Breast Milk

**DOI:** 10.1155/2024/1841909

**Published:** 2024-01-27

**Authors:** Naa N. Adumuah, Jude T. Quarshie, Harry Danwonno, Anastasia R. Aikins, Elmer N. Ametefe

**Affiliations:** West African Centre for Cell Biology of Infectious Pathogens (WACCBIP), Department of Biochemistry Cell and Molecular Biology, University of Ghana, Accra, Ghana

## Abstract

Current breast cancer treatment options are limited by drug resistance and adverse side effects, which calls for the need for alternatives or complementary remedies. Probiotic bacteria isolated from human breast milk have been shown to possess proapoptotic and anti-inflammatory properties against breast mastitis in breastfeeding mothers and are being studied as possible anticancer regimens. Thus, this study aimed at exploring the effect of lactic acid bacteria isolated from human breast milk on MDA-MB 231 breast cancer cells. A total of twenty-two bacteria were isolated from four human breast milk samples. The isolates were characterized and identified using biochemical tests and Sanger sequencing, respectively. For in vitro experiments, we used isolated *P. acidilactici* to treat MDA-MB-231 cells, and an MTT assay was used to detect proliferation. RT-qPCR and wound healing assays were performed to determine the effect of the isolated *P. acidilactici* on breast cancer cytokine expression and migration. Exposure of MDA-MB 231 breast cancer cells to live *P. acidilactici* and its cell-free supernatant (CFS) for 24 h resulted in a reduction in cancer cell viability. Also, the expression of the cytokines *IL-6*, *IL-8*, and *IL-10* in the breast cancer cells increased following exposure to *P. acidilactici* and its CFS for 24 and 72 h. Additionally, the levels of the *SLUG* gene remained unchanged while the *TWIST1* gene was upregulated following exposure of the cancer cells to bacteria, indicating that *P. acidilactici* may promote epithelial-mesenchymal transition in breast cancer. Finally, the CFS significantly inhibited cancer cell mobility. These findings serve as a foundation to further investigate the usefulness of *P. acidilactici* as a potential therapeutic agent in breast cancer therapy.

## 1. Introduction

Breast cancer is the most common cancer type and the leading cause of cancer-related deaths in women worldwide [1]. Despite the robust defence put up by the host immune system, breast cancer cells adapt several strategies to support their growth and survival. For example, breast cancer cells may secrete cytokines such as interleukin-6 (IL-6) and interleukin-8 (IL-8) which trigger signaling pathways involved in immune evasion, cell proliferation, angiogenesis, breast cancer chemoresistance, and epithelial-mesenchymal transition (EMT) [2].

EMT is a well-coordinated process in which cells lose their epithelial attributes like cell-cell contact and cell polarity to acquire mesenchymal attributes like high motility, poor differentiation, loss of cell-cell contacts, and hyperproliferation [3]. For EMT to be successful, there must be a downregulation of effectors such as E-cadherin, claudins, and occludins and the upregulation of vimentin, fibronectin, and N-cadherin. EMT core regulators include three main groups of transcription factors: the zinc finger E-box binding homeobox (ZEB) family (ZEB1 and ZEB2), the SNAIL family (SNAIL and SLUG), and the TWIST family (TWIST1 and TWIST2) [4]. These transcription factors are upregulated in breast cancer and are linked to invasiveness, metastasis, and poor prognosis [5].

The emergence of resistance to conventional therapies and other limitations of cancer treatment such as unpleasant side effects and high cost have prompted the need to explore alternatives for cancer therapy [6]. Emerging research into the use of probiotics as anticancer regimens is yielding positive results [7]. Probiotics are live organisms that confer health benefits on the host when ingested in reasonable amounts. They are abundant in fermented foods and drinks such as kombucha, kimchi, yogurt, and kefir [8]. Probiotics such as lactic acid bacteria (LAB) and bifidobacteria have been found to trigger immune responses, promote tumor necrosis, and possess antimicrobial activity [9]. Because they seem to have no detrimental effects on humans, probiotics are being considered as alternatives for anticancer therapy [10]. Indeed, some studies have proposed that ingesting lactobacilli and bifidobacteria may decrease the risk of developing certain cancers [11, 12]. Proposed mechanisms for the antitumor effects of probiotics include the secretion of antitumorigenic metabolites, neutralizing carcinogens, chelating heavy metals, and production of short-chain fatty acids [13].

Studies have reported that human breast milk contains a good amount of LAB within its microbiome [14, 15]. Other studies have demonstrated that several strains of probiotic bacteria isolated from human breast milk possess proapoptotic and anti-inflammatory properties against breast mastitis in breastfeeding mothers [16, 17]. Human breast milk-derived *Lactobacillus casei* and *Lactobacillus paracasei* strains also induce apoptosis in HeLa cells by downregulating antiapoptotic markers like Bcl-2 and upregulating proapoptotic genes like BAX, BAD, and caspases 3, 8, and c9 [18].

With the rapid adaptation and utilization of probiotics in modern medicine for the treatment of diseases, understanding the molecular processes of their action will enhance their applicability in the development of novel cancer approaches. This study isolated and characterized breast milk-derived LAB and investigated their anticancer properties in a breast cancer preclinical model.

## 2. Materials and Methods

### 2.1. Study Population

Four (4) lactating mothers were recruited for this study. Only apparently healthy lactating mothers who were not on medication for at least two months before the study were included in this study. Lactating mothers who showed any symptoms of disease or were on medication were excluded from the study.

### 2.2. Collection of Breast Milk Samples

The participants washed their hands, nipples, and areolas with soap and water before expressing about 5 mL to 10 mL of breast milk into autoclaved bottles provided. The milk samples were kept on ice and transported immediately to the laboratory.

### 2.3. Isolation of LAB

LAB from the samples was isolated as described by Rajoka et al. [15]. Briefly, the samples were serially diluted (10^−1^ to 10^−3^) with peptone water (Thermo Fisher Scientific, Carlsbad, CA, USA) and inoculated on de Man-Rogosa Sharpe (MRS) agar (Sigma-Aldrich, St Louis, MO, USA). The inoculated plates were incubated in anaerobic jars at 37°C for 48 h. Single colonies that grew were randomly selected, and each colony was inoculated in 5 mL of MRS broth (Sigma-Aldrich, St Louis, MO, USA) which was then incubated at 37°C for 48 h. The cultures were then streaked on MRS agar and incubated at 37°C for 48 h. Pure isolates were obtained and maintained at -80°C on MRS agar slants containing 40% glycerol pending further experiments.

### 2.4. Phenotypic and Biochemical Characterization of Isolated LAB

The isolates were characterized using a series of tests including gram staining, oxidase test, and catalase test as previously described [19].

### 2.5. Molecular Characterization of Isolated LAB

One (1) mL of the tentative LAB culture broth was centrifuged for 2 min at 14, 000 × g. The pellet was suspended in 200 *μ*L of sterile distilled water. DNA was extracted using a Quick-DNATM Fungal/Bacterial Miniprep Kit (Zymo Research, Irvine, CA, USA) per the manufacturer's protocol. The *16S rRNA* gene was amplified by polymerase chain reaction (PCR) using a 5PRIMEG/02 PrimeG Gradient Thermal Cycler. Thermocycling conditions were initial denaturation (98°C for 30 sec), 30 cycles of denaturation (98°C for 20 sec), annealing (62°C for 45 sec), and extension (60°C for 1.5 min). The amplified fragments were resolved on a 1.5% agarose gel for 90 min at 100 V. This was then visualized on the Amersham™ Imager 600. The PCR products were sequenced for identification by 16S rRNA Sanger sequencing.

### 2.6. Test for Probiotic Properties of Isolated LAB

Probiotics are characteristic for being able to survive in the human gut under conditions of relatively low pH, bile salt tolerance, and sensitivity to antibiotic treatment [15]. To test the growth of the isolates in low pH and high bile salt conditions, an acid tolerance test and bile salt tolerance were performed following published protocols [20] with slight modifications. Briefly, 100 *μ*L of overnight cultures were inoculated into 5 mL of MRS broth with pH adjusted to 3.0 using 0.1 M Hall (for acid tolerance) or 5 mL MRS supplemented with 0.3% of bile salt (for bile salt tolerance). The isolates were incubated at 37°C, and absorbance at a wavelength of 600 nm was measured hourly for 4 h. For antibiotic susceptibility testing, overnight cultures were spread on the Mueller-Hinton agar (Thermo Fisher Scientific, Carlsbad, CA, USA). Antibiotic-impregnated discs were then placed in quadrants on the plates, and zones of inhibition were measured after a 24 h incubation period at 37°C. The susceptibility of the bacteria isolates to antibiotics was evaluated according to the Clinical and Laboratory Standards Institute (CSLI) guidelines [21].

### 2.7. Cell Line and Culture

MDA-MB-231 breast cancer cell lines, obtained from the American Type Culture Collection, were maintained in Dulbecco's Modified Eagles Medium (DMEM) supplemented with 10% fetal bovine serum and 1% penicillin-streptomycin-glutamine (all purchased from Gibco, Life Technologies, Carlsbad, CA, USA) at 37°C in a humidified atmosphere containing 5% CO_2_.

### 2.8. Cell Treatments

The MDA-MB-231 cells were treated with isolated live *P. acidilactici* or its cell-free supernatant (CFS). One mL of overnight culture was centrifuged at 14,000 × g for 1 min and the supernatant was discarded. The pellet was washed twice with sterile phosphate buffer saline (PBS) and then suspended in 1 mL of DMEM to a working concentration of 10^6^ CFU/mL. To obtain CFS, the bacteria culture in DMEM was centrifuged at 14,000 × g for 1 min and the supernatant was collected and filtered through a 0.22 *μ*m Millipore filter.

### 2.9. Cell Viability Assay

The cells were seeded into 96-well plates at a density of 1 × 10^4^ cells/well and incubated at 37°C for 24 h. They were treated with 10 *μ*L of 10^4^, 10^5^, and 10^6^ CFU/mL of live *P. acidilactici* or its CFS for 24, 48, and 72 h. Then, 20 *μ*L of 2.5 mg/mL 3-(4,5-dimethylthiazol-2-yl)-2,5-diphenyltetrazolium bromide (MTT) (Sigma-Aldrich, St Louis, MO, USA) was added to each well and incubated at 37°C for 4 h. Afterward, 100 *μ*L of acidified isopropanol was added to each well and incubated at 37°C for 30 min. Absorbance was read at 570 nm with a Varioskan™ LUX multimode microplate reader (Thermo Fisher Scientific, Carlsbad, CA, USA). From the absorbance values, percent cell viabilities were calculated.

### 2.10. Wound Healing Assay

About 1 × 10^5^ cells/well were seeded into a 24-well plate and incubated at 37°C for 24 h. A sterile p200 pipette tip was used to create a wound in each well. The cells were treated with 10 *μ*L of 10^4^, 10^5^, and 10^6^ CFU/mL of live *P. acidilactici* or its CFS. Cell migration was monitored with an OPTIKA® microscope (OPTIKA, Ponteranica, Italy). The images were analyzed with ImageJ (NIH, Bethesda, MD, USA) to determine the percentage closure of the wound.

### 2.11. Reverse-Transcription Quantitative PCR (RT-qPCR)

About 1 × 10^6^ cells/mL of DMEM were seeded in 25 cm^2^ cell culture flasks for 24 h at 37°C. The cells were treated with 10 *μ*L of 10^6^ CFU/mL of live *P. acidilactici* or its CFS for 24, 48, and 72 h. The cancer cells were harvested, and total RNA was extracted using an RNeasy Mini Kit (Qiagen, Redwood City, CA, USA) per the manufacturer's protocol. Using a Luna Universal One-Step RT-qPCR Kit (New England Biolabs, Ipswich, MA, USA), RT-qPCR was performed to determine the mRNA expression of *IL-6*, *IL-8*, *IL-10*, *TWIST1*, and *SLUG* genes. A QuantStudio™ 5 RT-PCR System (Thermo Fisher Scientific, Carlsbad, CA, USA) was used for reverse transcription and amplification. Thermocycling conditions were reverse transcription (55°C for 10 min), initial denaturation (95°C for 1 min), 45 cycles of denaturation (95°C for 10 sec), annealing (different temperatures depending on the target gene for 15 sec), and extension (60°C for 1 min). The housekeeping gene *β-actin* was used as an internal control. Sequences and annealing temperatures of primers are listed in Supplementary Table S1. QuantStudio™ Design and Analysis Software (Life Technologies, Carlsbad, CA, USA) was used to obtain CT values, and the 2^−ΔΔCT^ method [22] was used to determine the relative expression of each gene.

### 2.12. Data and Statistical Analyses

Nucleotide reads from 16S rRNA sequencing were edited with the BioEdit 7.2.1 sequence edit analyzer. With the NCBI Basic Local Assignment Search Tool (BLAST), the organisms were identified based on sequence homology with other sequence databases. A phylogenetic tree was constructed with the MEGA X software [23]. Data were analyzed using GraphPad Prism 9.1.2 (GraphPad Software, San Diego, USA). One-way analysis of variance (ANOVA) followed by the Tukey post hoc test was used to compare differences between multiple groups. Data are presented as the mean ± standard deviation (SD) of at least three independent experiments performed in triplicates. Group differences were considered statistically significant when *p* < 0.05.

## 3. Results

### 3.1. Characterization of Breast Milk-Derived Bacteria

**Figure 1:**
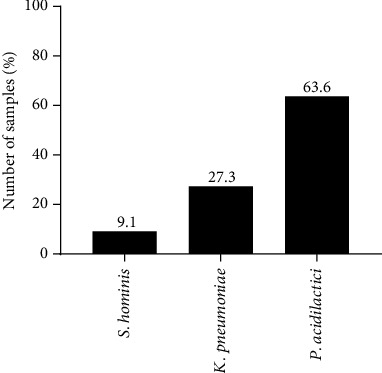
Percentage distribution of isolated LAB based on 16S rRNA sequencing analysis.

**Figure 2:**
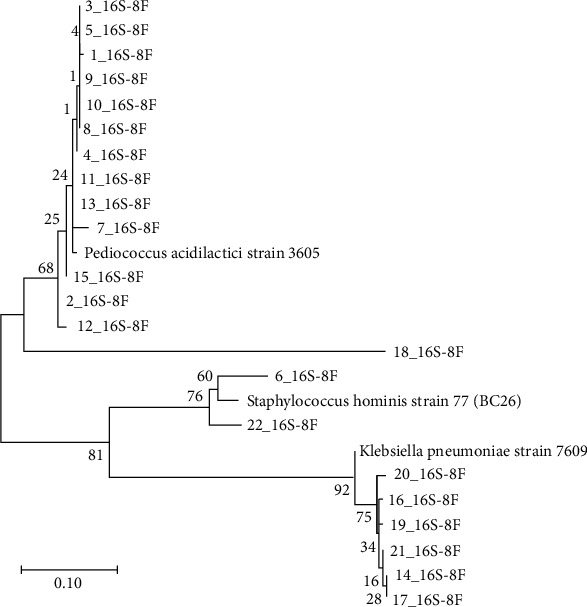
Phylogenetic diversity of isolated LAB. Numbers by nodes indicate levels of bootstrap support (%) based on analysis of 1000 resembled databases. The sequences were masked and aligned with the muscle maximum likelihood alignment.

A total of 22 single colonies were randomly selected from four different breast milk samples cultured on MRS broth and agar. Gram staining showed three distinct morphologies: Gram-positive diplococci, Gram-positive cocci, and Gram-negative bacilli (Supplementary Figure S1). The cocci and diplococci bacteria were catalase and oxidase-negative, whereas the bacilli were catalase-positive and oxidase-negative. The 22 isolates were further characterized using 16S rRNA Sanger sequencing after the full-length bacteria 16S rRNA gene was amplified by PCR and resolved on a 1.5% agarose gel electrophoresis (Supplementary Figure S2). Sanger sequencing showed that most of the isolates were *Pediococcus acidilactici*, followed by *Klebsiella pneumoniae* then *Staphylococcus hominis* (Figure 1). Phylogenetic analysis of the 22 isolates against the three species showed that most of the bacteria clustered with *P. acidilactici* (Figure 2).

### 3.2. Probiotic Potential of Isolated LAB

**Figure 3:**
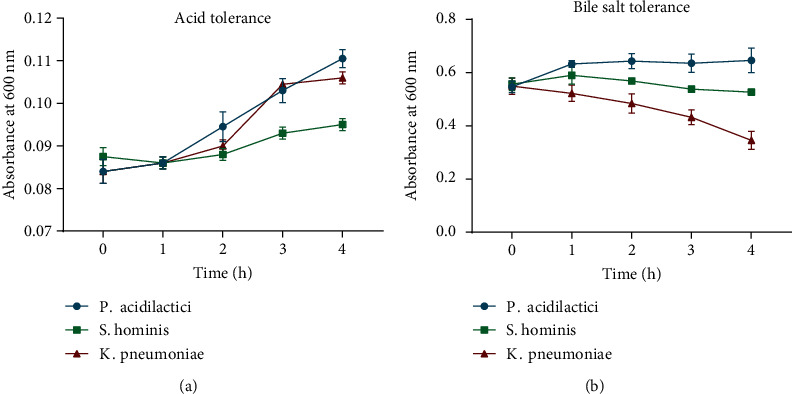
Probiotic potential of isolated LAB. (a) Acid tolerance and (b) bile salt tolerance of LAB. Bacteria were cultured in MRS broth with pH 3.0 or 0.3% bile salt, and absorbance was measured hourly for 4 h. Data are presented as mean ± SD of three independent experiments performed in triplicate.

The probiotic potential of the isolated LAB was investigated by evaluating their tolerance to acid and bile salt as well as their susceptibility to antibiotics. Generally, *P. acidilactici* was the most tolerant isolate as evidenced by a gradual increase in growth over a 4 h exposure period to acid and bile salt. *S. hominis* and *K. pneumoniae* showed a gradual increase in growth after exposure to acid. However, their growth slowly declined following exposure to bile salt (Figures 3(a) and 3(b)).

Probiotics are generally susceptible to antibiotics [15]. Nonetheless, the possibility of horizontal gene transfer among bacteria increases the tendency for probiotics to acquire antimicrobial resistance genes [24]. As such, it was important to determine the antibiotic profiles of the isolated LAB. The isolated *P. acidilactici* was susceptible to all the antibiotics tested, *S. hominis* was resistant to gentamycin, while *K. pneumoniae* was resistant to ampicillin, erythromycin, and kanamycin. (Table 1).

### 3.3. Effect of P. acidilactici and Its CFS on MDA-MB 231 Viability

**Figure 4:**
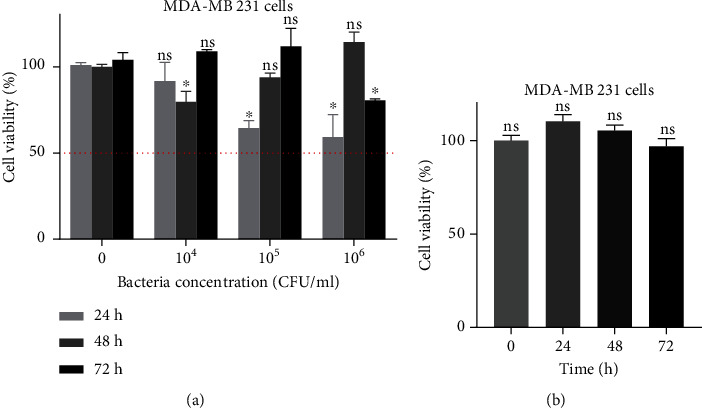
Effect of *P. acidilactici* and its CFS on MDA-MB 231 viability. MDA-MB 231 cells were treated with (a) live *P. acidilactici* or (b) CFS for 24, 48, and 72 h. Data are presented as mean ± SD of three independent experiments performed in triplicates. ^∗^*p* < 0.05 vs. untreated. ns: not significant.

Because *P. acidilactici* was the main LAB isolated from the sampled breast milk and it had the highest probiotic potential, we explored its anticancer effect. First, we examined the effect of live *P. acidilactici* or its CFS on MDA-MB 231 viability. Exposure of the MDA-MB 231 cells to the live bacteria for 24 h resulted in a dose-dependent decrease in cell viability, albeit not below 50% viability. Furthermore, exposure of the MDA-MB 231 cells to the live bacteria for 48 and 72 h caused variable, but insignificant changes in cell viability as bacteria concentration increased. On the other hand, treatment of MDA-MB 231 cells with the CFS had no significant effect on the cell viability (Figures 4(a) and 4(b)).

### 3.4. Effect of P. acidilactici and Its CFS on Cytokine Expression in MDA-MB 231 Cells

**Figure 5:**
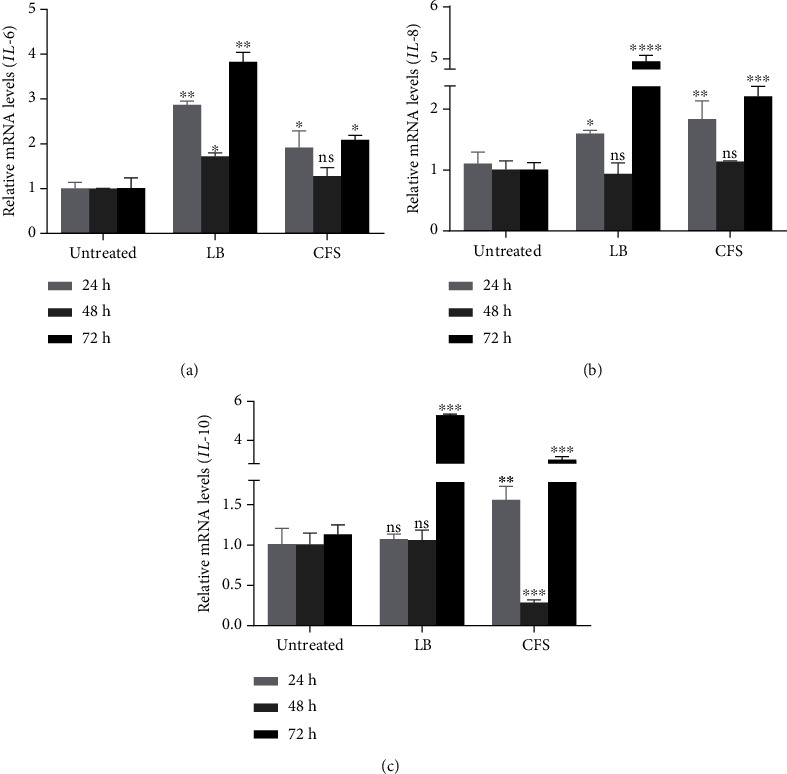
Effect of *P. acidilactici* and its CFS on cytokine expression in MDA-MB 231 cells. MDA-MB 231 cells were untreated or treated with live *P. acidilactici* (10^6^ CFU/mL) or CFS. (a) *IL-6*, (b) *IL-8*, and (c) *IL-10* mRNA levels were analyzed by RT-qPCR. Data are presented as mean ± SD of three independent experiments performed in triplicates. ^∗^*p* < 0.05, ^∗∗^*p* < 0.01, ^∗∗∗^*p* < 0.001, and ^∗∗∗∗^*p* < 0.0001 vs. untreated. LB: live bacteria; CFS: cell-free supernatant; ns: not significant.

Probiotic bacteria isolated from human breast milk have been shown to possess anti-inflammatory properties against breast mastitis in breastfeeding mothers [16, 17]; thus, we examined the effect of live *P. acidilactici* and its CFS on cytokine expression in MDA-MB 231 cells. We observed an upregulation of two proinflammatory cytokines, IL-6 and IL-8, after exposure of MDA-MB 231 cells to live *P. acidilactici* or CFS for 24 h or 72 h. Generally, the levels of these cytokines remained unchanged after 48 h of exposure to live *P. acidilactici* or CFS (Figures 5(a) and 5(b)). Although the levels of IL-10 (an anti-inflammatory cytokine) remained unchanged after 24 or 48 h of exposure to live *P. acidilactici*, there was an upregulation in IL-10 expression after 72 h of exposure. Furthermore, CFS induced IL-10 expression after 24 and 72 h, whereas it suppressed IL-10 expression after 48 h of treatment (Figure 5(c)).

### 3.5. Effect of P. acidilactici and Its CFS on EMT in MDA-MB 231 Cells

**Figure 6:**
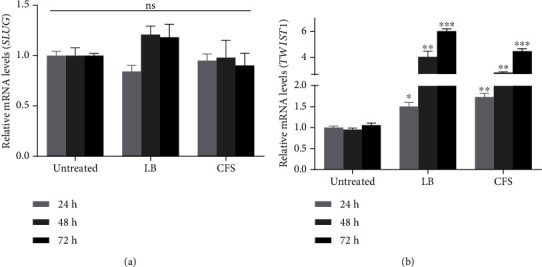
Effect of *P. acidilactici* and its CFS on EMT in MDA-MB 231 cells. MDA-MB 231 cells were untreated or treated with live *P. acidilactici* (10^6^ CFU/mL) or CFS. (a) *SLUG* and (b) *TWIST1* mRNA levels were analyzed by RT-qPCR. Data are presented as mean ± SD of three independent experiments performed in triplicates. ^∗^*p* < 0.05, ^∗∗^*p* < 0.01, and ^∗∗∗^*p* < 0.001 vs. untreated. LB: live bacteria; CFS: cell-free supernatant; ns: not significant.

Cytokines like IL-6 and IL-10 activate pathways involved in EMT via genes such as SLUG and TWIST1 [2]. To further probe the effect of *P. acidilactici* on breast cancer properties, we investigated the effect of the isolated bacteria on EMT in MDA-MB 231 cells. There were no significant changes in the expression of the *SLUG* gene in cells treated with live bacteria or CFS (Figure 6(a)). The expression of the *TWIST1* gene significantly increased with time in both the live bacteria and CFS-treated groups (Figure 6(b)).

### 3.6. Effect of P. acidilactici and Its CFS on MDA-MB 231 Cell Migration

**Figure 7:**
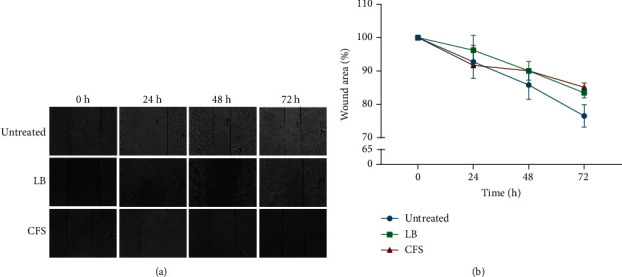
Effect of *P. acidilactici* and its CFS on MDA-MB 231 cell migration. MDA-MB 231 cells were untreated or treated with live *P. acidilactici* (10^6^ CFU/mL) or CFS. (a) Representative microscopy images (original magnification ×100) of migrating cells. (b) Wound area at time points is expressed as a percentage of the 0 h. Data are presented as mean ± SEM of three independent experiments performed in triplicates. LB: live bacteria; CFS: cell-free supernatant.

Next, we examined the impact of *P. acidilactici* and its CFS on cell migration using a wound healing assay. The wound area of the untreated control was reduced after 72 h. Although live *P. acidilactici* had no significant effect on wound closure, wound area in the CFS-treated group was higher compared to untreated cells. This signifies that the CFS negatively influences MDA-MB 231 cell migration (Figure 7).

## 4. Discussion

Human breast milk possesses a unique microbiome consisting of a wide range of LAB belonging to *streptococci*, *staphylococci*, *lactobacilli*, *enterococci*, and *micrococci* species [25]. Evidence suggests that some of these microbes may complement or be alternatives in standard cancer therapy. However, the mechanisms of their action are yet to be fully understood. Here, a combination of phenotypic, biochemical, and molecular techniques was employed to characterize LAB present in the breast milk of healthy lactating mothers, and the anticancer properties of breast milk-derived *P. acidilactici* were evaluated in a breast cancer model.

In this study, we isolated three main LAB from the sampled breast milk, namely, *S. hominis*, *P. acidilactici*, and *K. pneumoniae*. This is consistent with earlier research which found *S. hominis* and *P. acidilactici* in the breast milk of healthy lactating mothers [17, 26]. The isolated *S. hominis* and *P. acidilactici* survived high acid and bile salt conditions, indicating that they possess the ability to survive the harsh environment of the human gut as probiotics. *K. pneumoniae* was unable to tolerate the harsh treatment of bile salts, suggesting that it may not be adapted to colonize the stomach or small intestines of the human gut. Furthermore, the isolated LAB were generally susceptible to all antibiotics, further confirming their probiotic properties.

Research has demonstrated that some LAB and their metabolites inhibit cancer progression by reducing cell viability, survival, and angiogenesis. For example, *Lactobacillus* sp., *S. hominis*, and *E. faecalis* have been shown to decrease the viability of breast, cervical, and colon cancer cell lines [17, 18, 27, 28]. In this study, the viability of MDA-MB 231 cells decreased in a dose-dependent manner after 24 h of exposure to live *P. acidilactici*. Exposure for 48 and 72 h resulted in an irregular trend in cell viability which was generally insignificant. This is similar to reports by Hassan et al., who observed a dose-dependent decrease in the viability of MCF-7 breast cancer cells after 24 h of exposure to live *E. faecalis* and an unexpected increase in viability after 48 and 72 h of exposure [17]. The trend we observed could be attributed to the loss of bacteria viability over time, resulting in minimal cell interaction or lowered release of metabolites necessary to inhibit cancer cell growth. Treatment of MDA-MB 231 cells with *P. acidilactici*-derived CFS did not affect cell viability, suggesting that direct contact between the cancer cells and bacteria is required for cytotoxicity. This is substantiated by the theory that adhesion to human epithelial cells is a key prerequisite for probiotic action [29].

Proinflammatory cytokines IL-6 and IL-8 and anti-inflammatory cytokine IL-10 play vital roles in breast cancer progression. They are expressed by cancer cells or by immune and stromal cells within the tumor microenvironment. IL-6 and IL-8 are involved in proinflammatory responses, angiogenesis, and stemness and are negative prognostic markers for breast cancer [2, 30]. On the other hand, the role of IL-10 in breast cancer is still unclear. While some studies have shown that IL-10 inhibits tumorigenesis, others have shown a correlation between high levels of IL-10 and an increased propensity for cancer cell survival and immune evasion [31, 32]. In this study, the expression of *IL-6*, *8*, and *10* were significantly higher in the MDA-MB 231 cells treated with live *P. acidilactici* and its CFS than in the untreated cells, indicating that exposure of the cancer cells to bacteria triggered inflammatory responses that promote cancer progression. This observation could be due to contact of the cancer cells with bacterial cell wall structures like lipoteichoic acid and peptidoglycans which induce cytokine expression via toll-like receptor activation [33].

The upregulation of IL-6, 8, and 10 results in the activation of the STAT3 protein which then activates EMT-associated genes such as *TWIST1* and *SLUG* [34–36]. Here, we observed a time-dependent increase in the expression of *TWIST1* after exposure to live bacteria or CFS, whereas no significant change was seen in the expression of *SLUG*. This suggests that exposure of MDA-MB 231 cells to live *P. acidilactici* and its CFS may enhance EMT and as such tumor progression. Again, we found that the CFS reduced cancer cell motility. Based on our initial observation that the CFS had no significant effect on cell viability after 72 h, it can be confirmed that the bacteria CFS indeed reduces cancer cell motility, which is a favorable outcome.

## 5. Conclusions

Our study provides preliminary evidence of the anticancer potential of human breast milk-derived *P. acidilactici* and its metabolites. Their presence caused the upregulation of proinflammatory cytokines and the *TWIST1* gene involved in EMT. Furthermore, *P. acidilactici* metabolites reduced the motility of the cancer cells. Our findings serve as foundational work to further investigate the usefulness of *P. acidilactici* as a potential therapeutic agent in cancer therapy.

## Figures and Tables

**Table 1:** Antibiotic susceptibility test for breast milk-derived bacteria.

Antibiotic	Disc content (*μ*g/mL)	*P. acidilactici*	*S. hominis*	*K. pneumoniae*
Ampicillin	10	S	S	R
Chloramphenicol	30	S	S	S
Erythromycin	15	S	S	R
Gentamycin	10	S	R	S
Kanamycin	30	S	S	R
Streptomycin	10	S	S	S
Tetracycline	30	S	S	S

S: susceptible; R: resistant.

## Data Availability

All data are presented within the article.

## Supplementary Materials

Supplementary Materials Supplementary Table S1: sequences and annealing temperatures of primers used. Supplementary Figure S1: gram staining showing Gram-positive diplococci, Gram-positive cocci, and Gram-negative bacilli. Supplementary Figure S2: agarose gel showing PCR-amplified 16S rRNA gene band from some bacteria bacterial isolates.
