# Supplementary material for: Exploring Anti-Breast Cancer Effects of Live Pediococcus acidilactici and Its Cell-Free Supernatant Isolated from Human Breast Milk
Source: Int J Breast Cancer. 2024 Jan 27;2024:1841909. doi: 10.1155/2024/1841909 (PMC10838206; doi:10.1155/2024/1841909)
Supplement: Supplementary Materials — Supplementary Table S1: sequences and annealing temperatures of primers used. Supplementary Figure S1: gram staining showing Gram-positive diplococci, Gram-positive cocci, and Gram-negative bacilli. Supplementary Figure S2: agarose gel showing PCR-amplified 16S rRNA gene band from some bacteria bacterial isolates. [file 1841909.f1.docx]

**Exploring Anti-Breast Cancer Effects of Live *Pediococcus acidilactici* and its Cell-Free Supernatant Isolated from Human Breast Milk**

Naa N. Adumuah, Jude T. Quarshie, Harry Danwonno, Anastasia R. Aikins, and Elmer N. Ametefe

West African Centre for Cell Biology of Infectious Pathogens (WACCBIP), Department of Biochemistry Cell and Molecular Biology, University of Ghana, Accra, Ghana

Corresponding author

Elmer Nayra Ametefe: eametefe@ug.edu.gh

Table S1: Sequences and annealing temperatures of primers.

| Gene | Sequence | Annealing temp. (°C) |
| --- | --- | --- |
| *16S rRNA* | pA AGAGTTTGATCCTGGCTCAG | 62.0 |
|  | pH AAGGAGGTGATCCAGCCGCA |  |
| *IL-6* | 5’-GCCCAGCTATGAACTCCTTCT-3’ | 56.0 |
|  | 5’-GAAGGCAGCAGGCAACAC-3’ |  |
| *IL-8* | 5’-GTGCAGTTTTGCCAAGGAGT-3’ | 56.0 |
|  | 5’-TTATGAATTCTCAGCCCTCTTCAAAAACTTCTC-3’ |  |
| *IL-10* | 5’- GGTTGCCAAGCCTTGTCTGA-3’ | 56.0 |
|  | 5’-AGGGAGTTCACATGCGCCT3’ |  |
| *TWIST1* | 5’-GGGAGTCCGCAGTCTTAC-3’ | 53.5 |
|  | 5’-CCTGTCTCGCTTTCTCTTT-3’ |  |
| *SLUG* | 5’-GGTCAAGAAGCATTTCAAC-3’ | 53.5 |
|  | 5’-GGTAATGTGTGGGTCCGA-3’ |  |
| *β-actin* | 5’-TTCCTGGGCATGGAGTCCTGTGG-3’ | 56.0 |
|  | 5’-CGCCTAGAAGCATTTGCGGTGG-3’ |  |


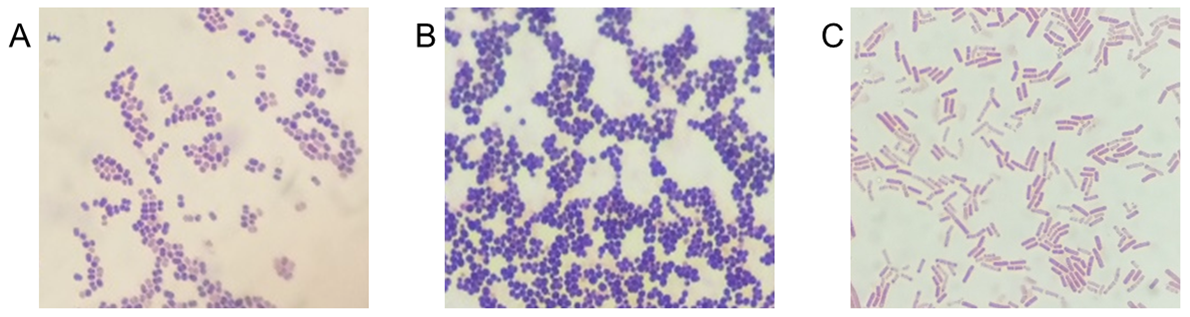


**Supplementary Fig. S1** Morphological identification of isolates. Microscopy images show (**A**) diplococci, (**B**) cocci and (**C**) bacillus bacteria. Gram positive or negative bacteria are stained purple or pink, respectively. Original magnification ×100


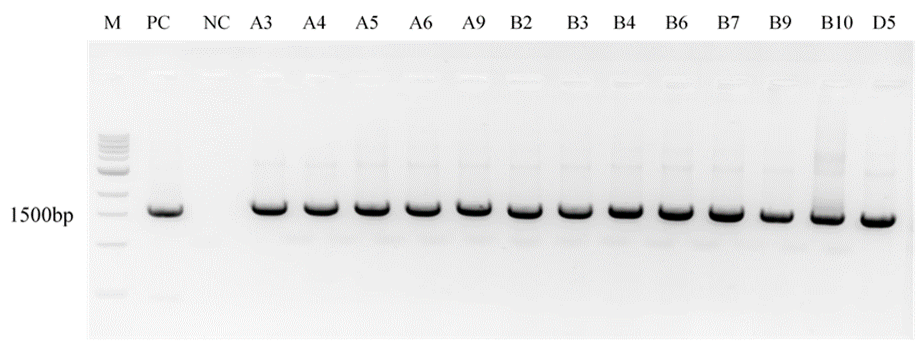


**Supplementary Fig S2** PCR amplification of *16S rRNA* gene in isolated LAB. Agarose gel showing amplified *16S rRNA* gene with band size of 15kbp for thirteen of the twenty-two isolates labelled A3 and D5. M represents the 1Kb molecular ladder, PC: positive control, NC: negative control
